# Immunohistochemical and immunocytochemical study of mechanoreceptors in anterior cruciate ligament reconstruction with the remnant-preserving technique using Achilles tendon allografts

**DOI:** 10.1186/s13018-017-0593-0

**Published:** 2017-06-14

**Authors:** Keun Churl Chun, Sung Hyun Lee, Jeong Woo Kim, Eun Jung Jin, Kwang Mee Kim, Churl Hong Chun

**Affiliations:** 1 Department of Orthopedic Surgery, School of Medicine, Wonkwang University Hospital, Muwang-ro 895, Iksan-si, Jeolabuk-do South Korea; 2 Naval Hospital of Korean Armed Forces, Pohang, South Korea; 3 Department of Biological Sciences, College of Natural Sciences, Wonkwang University, Iksan-si, South Korea; 4 Department of Nursing, Chodang University, Muan, South Korea

**Keywords:** Anterior cruciate ligament, Remnant-preserving technique, Immunohistochemical study of mechanoreceptor, Immunocytochemical study of mechanoreceptor

## Abstract

**Background:**

Attempts have been made to validate the significance of remnant preservation with anterior cruciate ligament (ACL) reconstruction using immunohistochemical and immunocytochemical techniques. The purpose of this study was to examine the expression of mechanoreceptors in the remnant tissue of ACL reconstruction performed with the remnant-preserving technique.

**Methods:**

Tissue samples were obtained from 10 patients who underwent ACL reconstruction with the remnant-preserving technique. The specimens were obtained from remnant ACL tissue and Achilles allografts superficially and at the tibial attachment. The control group consisted of three normal ACLs procured from young males who underwent partial meniscectomy. Tissues and cells from the ACL remnants and Achilles allografts were characterized using hematoxylin and eosin (H&E) staining and immunohistochemical, immunocytochemical, and immunoblotting assays. In particular, the sensitivity of neural cell validation was improved using nerve growth factor (NGF) to stimulate the expression of neural cells.

**Results:**

The results are summarized as follows. (1) In H&E staining and immunohistochemical assays, no neural cells were detected in remnant or allograft tissue. (2) In the immunocytochemical study, neural cells were detected in remnant tissue. (3) The increased proliferation of remnant ACL cells with NGF treatment suggested their identity as neural cells. (4) NGF treatment also stimulated protein and RNA expression of Nestin (a specific marker for neural cells) in remnant ACL cells.

**Conclusions:**

The improved immunocytochemical methodology proved useful. Although mechanoreceptors were detected relatively less frequently than expected, the authors consider that this finding does not negate the necessity of remnant-preserving ACL reconstruction.

## Background

Injury to the anterior cruciate ligament (ACL) is one of the most common causes of knee instability and resultant disability in sports. Deficiency of the ACL often causes repeated episodes of instability, meniscal tearing, and osteochondral injuries, which can eventually lead to arthritis [15]. Barrett [3] proposed that the functional instability that occurs after injury to the ACL is due to the combined effects of excessive tibial translation and lack of coordinated muscle activity to stabilize the knee joint. Even when the mechanical integrity has been restored after ACL reconstruction performed on highly unstable patients, some reconstructions still show functional instability during sports activities.

Hogervorst and Brand [8] indicated that it is important to restore not only mechanical function but also proprioception by mechanoreceptors, which is related to functional stability. Thus, mechanoreceptors have generated significant interest. Indeed, several authors have studied mechanoreceptors in the remnant stumps of injured ACLs [2, 7, 13].

However, there is still no consensus as to whether the remnant technique is clinically superior to the traditional technique. Some papers report that the currently available evidence is not sufficiently strong to support the superiority of remnant-preserving ACL reconstruction [11, 16, 20]. Muneta et al. [16] reported that remnant volume was weakly correlated with postoperative outcome regarding objective stability and subjective recovery. Park et al. [19] also reported that remnant-preserving augmentation and double-bundle reconstruction showed similar results in terms of anterior and rotary stability and clinical scores.

In contrast, some authors have reported that remnant preservation techniques for ACL reconstruction preserve proprioception and lead to positive results [1, 12, 18, 21]. Takazawa et al. [21] confirmed that preserving the remnant tissue of the ACL could facilitate recovery of function and decrease graft rupture after primary reconstruction. Kase et al. [9] noted that it is important to preserve the bridging anatomical insertions of the ACL on the lateral wall of the femoral condyle and the tibia when ACL reconstruction surgery is performed.

A clinical approach to assess technique success is important, although histological analysis should also be performed when investigating the remnant ACL reconstruction technique [4]. A few authors have reported remnant preservation techniques of ACL reconstruction to preserve mechanoreceptors with positive results [1, 12, 18]. However, most previous studies histologically evaluated the presence of mechanoreceptors at initial ACL reconstruction. One previous study histologically evaluated the presence of mechanoreceptors in remnant ACL tissue and allografts in second-look arthroscopy after ACL reconstruction; however, there was no evidence of newly ingrown mechanoreceptors via histological examination [10]. It is important that mechanoreceptors persist after remnant preservation ACL reconstruction, although whether these receptors serve as a source of re-innervation or proprioception is not clear. Therefore, improving upon the technique of using nerve growth factor (NGF) to stimulate the expression of neural cells, we sought to validate and document the significance of the remnant technique. The purpose of this study was to investigate the histological existence of mechanoreceptors and the cytological existence of nerve cells in the allograft and remnant site of ACL reconstruction following the remnant-preserving technique. Our hypothesis was that remnant tissue following ACL reconstruction would contain mechanoreceptors, which was demonstrated by our immunohistochemical and immunocytochemical analysis.

## Methods

This study was conducted in compliance with the principles derived from the Declaration of Helsinki and was approved by the institutional review boards of the Hospital Ethics Committees. All patients signed the informed consent form, and the project was approved by the Regional Ethical Committee.

Among the patients who underwent ACL reconstruction using the remnant-preserving technique from January 2011 to December 2012, 10 patients were randomly selected for second-look arthroscopy for investigational purposes (Table 1). Second-look arthroscopy was performed to observe whether the ACLs were successfully reconstructed after removing the screw and washer. Two patients were in their teens (two males; mean age, 19 years), five were in their 20s (four males, one female; mean age, 23.5 years), two were in their 40s (two males; mean age, 43 years), and one was in her 50s (one female, 58 years). The mean age of the patients at the time of biopsy was 29.7 years (range, 18~58 years). The mean period from first injury to reconstruction was 5.1 weeks (range, 2~16 weeks). The mean period from ACL reconstruction to harvesting of the tissue was 20.9 months (range, 13~31 months).

**Table 1:** Data on patients and number of specimens

No.	Age/sex	Duration from injury (weeks)^a^	Period (month)^b^	Lysholm score
1	22/F	4	15	95
2	58/F	12	13	96
3	25/M	2	16	97
4	45/M	4	31	98
5	41/M	5	50	95
6	29/M	4	15	95
7	19/M	2	26	95
8	20/M	9	43	98
9	20/M	16	40	96
10	18/M	2	17	95

^a^Duration from injury to ACL reconstruction

^b^Period between ACL reconstruction and harvesting of tissue

Single-bundle ACL reconstruction was performed, and the femoral tunnel was prepared with the trans-tibial technique at the 10:30 and 1:30 clockface positions.

**Fig. 1:**
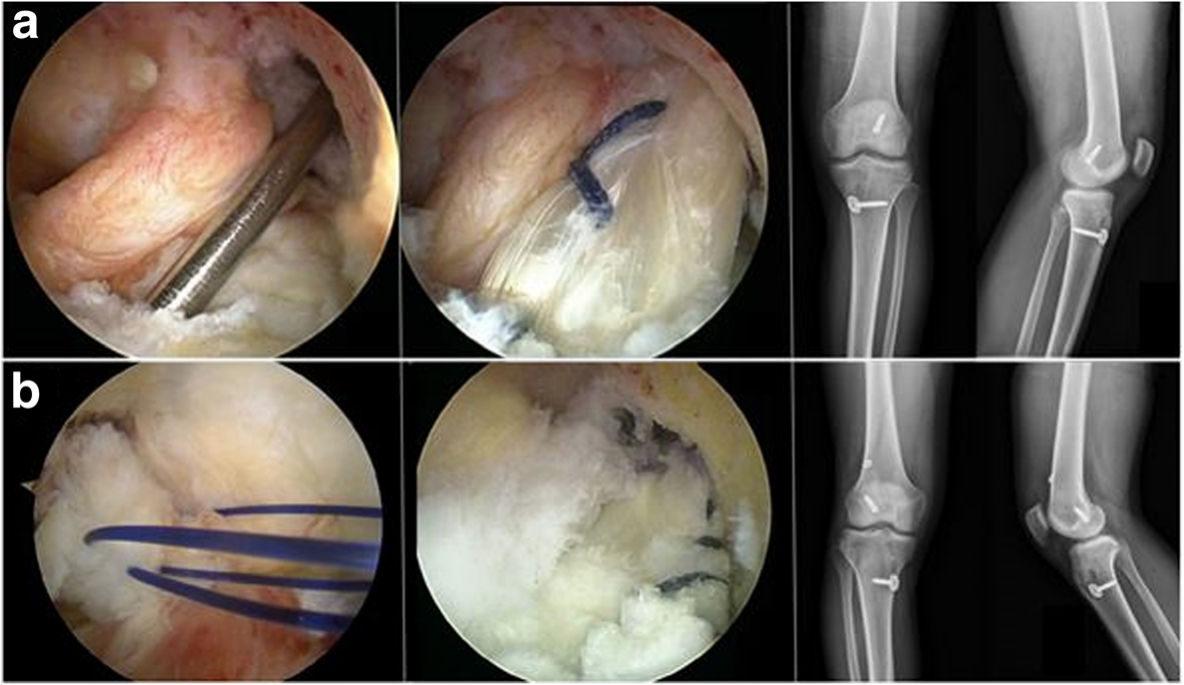
Arthroscopic findings for both types of preservation technique with postoperative radiography. **a** The remnant preservation technique with Achilles allograft reconstruction. **b** The remnant suture and tensioning technique with Achilles allograft reconstruction

The remnant preservation technique with Achilles allograft reconstruction was performed in five patients. The remnant lesion of the torn ACL was noted to be of adequate length, with natural tension and truncated shape. The Achilles tendon allograft was passed laterally at the remnant site through the tibial to femoral tunnel (Fig. 1a). The remnant suture and tensioning technique with Achilles allograft reconstruction was performed in the other five patients. In these patients, the remnant of the torn ACL was sutured to the allograft with three to four bundles of polydioxanone. In all patients, the graft was tensioned and fixed through the femoral tunnel using an endo-button (Arthrex, Naples, FL, USA) (Fig. 1b).

Among the cases, there was one case of combined medial collateral ligament (MCL) injury, and the authors treated the MCL injury non-operatively. In two cases of combined meniscal injury, the authors performed partial meniscectomy. Conventional ACL rehabilitation protocols progressing to full range of motion (ROM) and full weight bearing by 6 weeks were used in all cases.

**Fig. 2:**
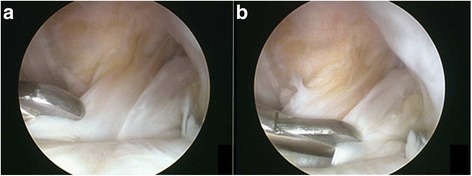
Specimens were obtained at the tibial ACL reconstruction site of **a** remnant ACL tissue and **b** Achilles allograft tissue by second-look arthroscopy

Biopsies were performed using a punch biopsy instrument at the time of second-look arthroscopy. The size of the remnant harvested ranged from 0.3 to 0.6 cm^2^ (mean 0.43 cm^2^). The ACL harvest sites were the tibial site of remnant ACL tissue and the allograft (Fig. 2). A total of 20 specimens were obtained, 10 from the remnant preservation site and 10 from the Achilles allograft site. The control group consisted of three normal ACLs procured from 20-, 25-, and 31-year-old men who underwent partial meniscectomy after providing informed consent. In the control group, biopsies were performed on the tibial side of the normal ACL using a punch biopsy instrument.

To investigate the possible existence of neuronal cells, immunohistochemical and immunocytochemical analyses were applied.

### H&E staining and immunohistochemical staining

Specimens were obtained from the remnant tissue and allografts. Tissue slices were fixed with masked formalin solution (masked form 2A, DANA Korea) for 24 h and then embedded in paraffin after dehydrating with alcohol and clearing with a tissue processor (Leica TP 1020, Leica, Germany). The specimens were sequentially sliced at a thickness of 4 μm and stained with hematoxylin and eosin (H&E).

The authors used a Fully Automatic IHC & ISH Staining System (Ventana Medical System, Inc., USA) for immunohistochemical assessment. The primary antibody for this study was rabbit anti-cow S-100 (2000:1, Dako, Denmark; cat. no. Z0311). The detection kit was the UltraView DAB Detection kit (Ventana Medical System, Inc., AZ, USA), which included the DAB inhibitor, HRP multimer, DAB chromogen, H_2_O_2_, and copper.

The samples were embedded in deparaffinization solution. Incubation with collagen antibody was performed for 32 min at 42 °C in a humidified chamber. After counterstaining with hematoxylin-1 and Bluing (Ventana Medical System, Inc., AZ, USA; cat. no. 760-2021) for 4 min, the samples were dehydrated and mounted in Synthetic Mount (Shandon, Pittsburgh, USA; cat. no. 6769007).

According to Freeman and Wyke, mechanoreceptors are classified into four types: type I, as a spherical or ovoid Ruffini corpuscle; type II, as a columnar concentric circular Pacini corpuscle; type III, as a spindle-shaped Golgi corpuscle; and type IV, as a non-myelinated free nerve ending.

### Immunocytochemical assay

#### Cell culture and treatments

Ligament tissue was rinsed with saline, followed by incubation with trypsin (Gibco, Life Technologies, Paisley) at 37 °C for 10 min. After removing the trypsin solution, the ligament slices were treated for 6–8 h with type IV clostridial collagenase in Dulbecco’s modified Eagle’s medium (DMEM, Gibco Invitrogen, Grand Island, NY) with 10% FBS (Gibco Invitrogen, Grand Island, NY) to release the ligament cells. The cells were maintained in culture medium for the indicated time periods in the presence or absence of 50 ng/ml NGF (R&D Systems, Minneapolis, MN).

#### Cell proliferation assay

Proliferation of cells was determined by direct counting of cells from cultures. Control and NGF-treated cultures were detached with trypsin/EDTA solution and counted in triplicate using a cell counter (Countess, Invitrogen, Grand Island, NY). Images of stained cells were captured (two images/experiment), and different areas (square areas) were selected for counting.

#### Immunofluorescence

Cells grown on coverslips were washed three times with phosphate-buffered saline (PBS), fixed with 4% paraformaldehyde in PBS for 10 min, washed three times with PBS, and then permeabilized with 0.1% Triton X-100 in PBS for 5 min at room temperature. After washing three times in PBS, cells were blocked with 1% bovine serum albumin (BSA) for 1 h at room temperature. Incubation with anti-Nestin antibody (Sigma, St. Louis, MO) was performed in blocking solution (1% BSA in PBS) for 1 h at room temperature in a light-proof box. Specimens were washed three times with PBS and incubated for 1 h at room temperature with Alexa Fluor 555-conjugated rabbit anti-goat antibody (Invitrogen, Grand Island, NY).

For F-actin staining, Alexa Fluor 488-conjugated phalloidin (Invitrogen, Grand Island, NY) was used. Nuclei were stained with 4,6-diamidino-2-phenylindole (DAPI, Santa Cruz Biotechnologies, Santa Cruz, CA). A confocal microscope (Olympus, FV-1mm) was used for capturing images.

#### Western blotting

Cells were lysed in RIPA buffer, and the protein concentration of cell lysates was determined with a bicinchoninic acid protein assay (Pierce Biotechnology Inc., Rockford, MN). Thirty micrograms of protein was separated by electrophoresis on 10% polyacrylamide gels containing 0.1% sodium dodecyl sulfate (SDS) for 10% SDS PAGE, and proteins were transferred to nitrocellulose membranes (Whatman, Piscataway, NJ). The membranes were incubated for 1 h at room temperature in blocking buffer (20 mM Tris-HCl, 137 mM NaCl, pH 8.0, containing 0.1% Tween and 3% non-fat dry milk) and incubated with anti-Nestin antibody (Sigma, St. Louis. MO). Then, the membranes were incubated with a peroxidase-conjugated secondary antibody for 1 h at room temperature, and the signals were detected using an enhanced chemiluminescence (ECL) system (Pierce Biotechnology Inc., Rockford, MN).

#### Quantitative real-time polymerase chain reaction

**Fig. 3:**
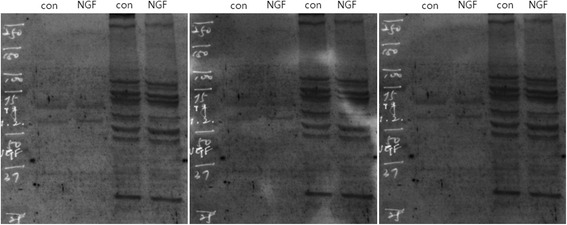
Quantitative RT-PCR was performed to assess the expression of nerve growth factor (NGF). Specific primers (5′-forward 5′-GTCATCATCCCATCCCATCTTC-3′, reverse 5′-CACACACAGGCCGTATCTATC-3′) were applied, and the transcription levels of GAPDH were used as a reference

Transcripts of nerve growth factor were quantified by RT-PCR using specific primers (5′-forward 5′-GTCATCATCCCATCCCATCTTC-3′, reverse 5′-CACACACAGGCCGTATC TATC-3′) and normalized to the amount of GAPDH mRNA expressed. The thermal cycling conditions were as follows: 1 cycle at 95 °C for 10 min; 30 cycles at 94 °C for 15 s, 55 °C for 30 s, and 72 °C for 30 s; and 1 cycle at 72 °C for 10 min (Fig. 3).

## Results

### Hematoxylin-eosin staining and S-100 staining

**Fig. 4:**
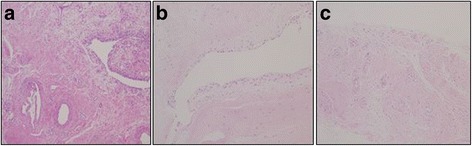
In all cases, synovium with good synovial and intrafascicular vascularity was seen. **a** Normal ACL (control specimen). **b** Remnant specimens. **c** Achilles allograft specimens (H&E stain, ×100)

**Fig. 5:**
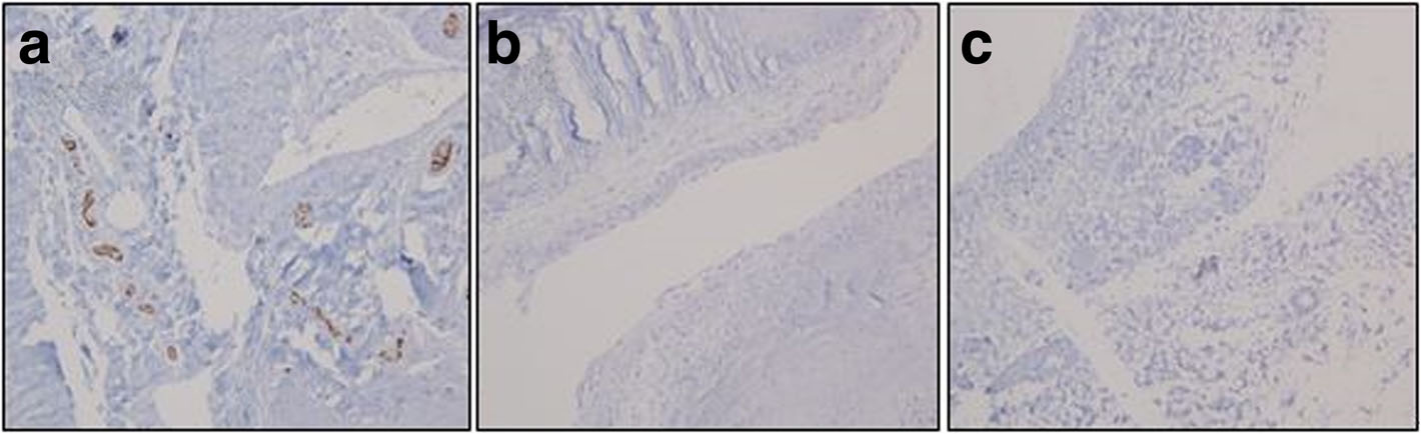
Staining with a monoclonal antibody against S-100 showing intraligamentous mechanoreceptors (*brown color*) in **a** normal ACL tissue (control group), **b** remnant specimens, and **c** Achilles allograft specimens (immunohistochemical stain, ×200)

H&E examination confirmed ligamentous ACL tissue in all specimens. Synovium with good synovial and intrafascicular vascularity was seen in all cases (Fig. 4). Monoclonal antibodies against S-100 were used to stain Schwann cells and nerve tissues. Ruffini corpuscles and Pacini corpuscles were observed in the control group when nervous tissues were classified morphologically. In controls, many S-100-positive cells were seen. However, none of the remnant ACL tissues or Achilles allograft tissues displayed any S-100-positive cells (Fig. 5). After rehydrating the Achilles allograft, which was fresh-frozen before surgery in a warm saline solution mixed with gentamicin for 30 min, light microscopic examination of the specimens collected from the allograft showed no cells (Fig. 5).

### Expansion of cells in ACL and Achilles allograft tissues

**Fig. 6:**
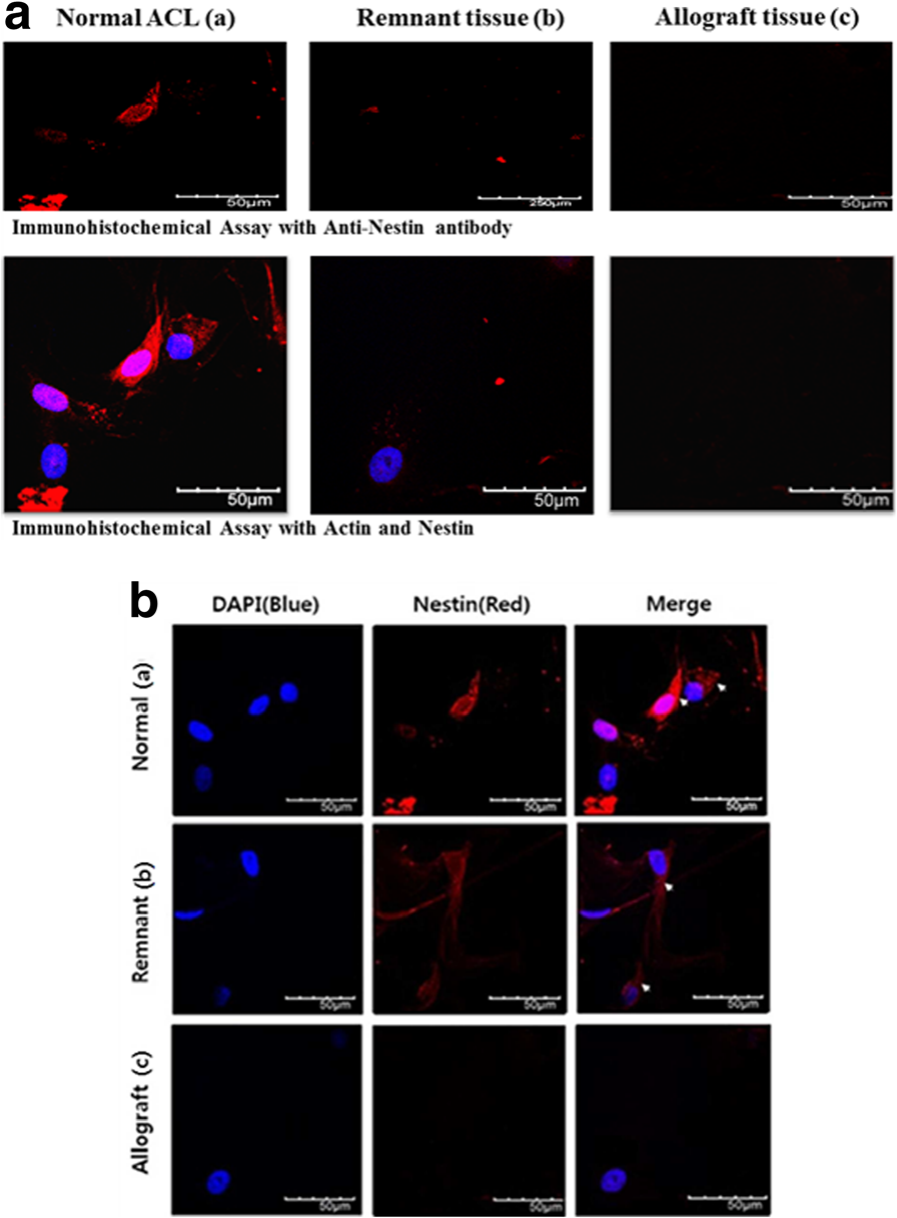
**A** Immunochemical assay with an anti-Nestin antibody in *(a)* normal ACL tissue, *(b)* remnant specimens, and *(c)* Achilles allograft specimens. **B** Immunocytochemical characterization of nerve cells. Nuclei were stained with 4,6-diamidino-2-phenylindole (DAPI, Santa Cruz Biotechnologies, Santa Cruz, CA). Nerve cells stained with an anti-Nestin antibody in *(a)* normal ACL tissue, *(b)* remnant specimens, and *(c)* Achilles allograft specimens

**Fig. 7:**
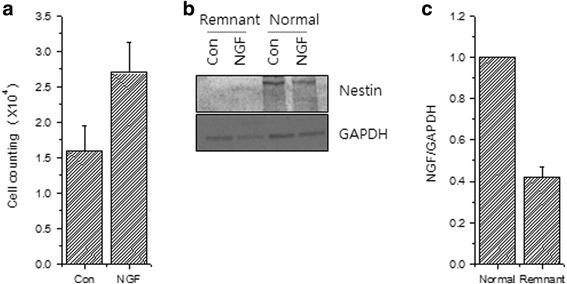
**a** Cell proliferation assay using cells isolated from remnant tissues of reconstructed ACLs and treated with NGF. **b** Protein expression of Nestin. **c** RNA expression of NGF

The presence of neuronal cells was not detected in either remnant ACL or Achilles allograft tissues from reconstructed ACL patients based on H&E staining (Fig. 5). In addition, in four cases, immunocytochemical analysis of remnant tissue using the anti-Nestin antibody showed no Nestin-positive cells (patient nos. 2, 4, 5, 9 in Table 1). However, six remnant ACL tissues showed few Nestin-positive cells, suggesting the possible existence of neuronal cells in the remnant ACL tissue (Fig. 6). The expression level of Nestin in remnant ACL tissue cells was also relatively low (40% of control) compared with that in normal cells (Fig. 7b).

For further verification, we cultured cells with NGF, a well-known growth factor for nerve cell proliferation. We observed an increase in the proliferation of remnant ACL tissues with NGF treatment (Fig. 7a). Moreover, the translation (Fig. 7b) and transcription (Fig. 7c) of Nestin as analyzed by immunoblotting confirmed the existence of neuronal cells. While Nestin protein was detected in remnant ACL tissue with a relatively weak signal compared with that in normal ACL tissue, this expression increased with NGF treatment in remnant ACL tissue (Fig. 7b).

## Discussion

Twenty biopsy specimens were obtained from 10 patients at the time of second-look arthroscopy following ACL reconstruction using allograft tendon grafts with the remnant preservation technique. These specimens were characterized using H&E staining and immunohistochemical, immunocytochemical and immunoblotting assays after cell culture for 6 weeks. The important findings in this study are listed as follows. First, in H&E staining and immunohistochemical assays, no neural cells were detected in the remnant or allograft tissue. Second, in the immunocytochemical study, neural cells were detected in the remnant tissue. Third, increased proliferation of remnant ACL cells was observed upon NGF treatment, suggesting the identity of these cells as neural cells. Moreover, the protein and RNA expression of Nestin (a specific marker of neural cells) was also observed in remnant ACL cells.

Proprioception is a major factor assisting knee joint stability following functional deficiencies in sports activities after restoration of mechanical integrity with ACL reconstruction. Ochi et al. [17] emphasized that restoration of knee function is not only important in terms of the anatomical ACL reconstruction as a mechanical restraint but also in terms of sensory re-innervation of the graft, which could potentially improve overall outcomes. Denti et al. [5] detected re-innervation of autologous bone-patellar tendon bone grafts in animals 3–6 months postoperatively. Several authors have studied mechanoreceptors in the remnant stumps of injured ACLs [2, 7, 13], and a few authors have shown that remnant preservation techniques for ACL reconstruction preserve mechanoreceptors, with positive results [1, 18]. Therefore, we investigated the expression of mechanoreceptors in remnant tissues and allografts following remnant-preserving ACL reconstruction.

There have been many studies investigating proprioception and kinesthesia as well as the function of mechanoreceptors in reconstructed ACLs. However, it is difficult to compare results between studies because experimental methods are variable and many external factors influence the results. Therefore, we performed histological examinations and cytochemical assays. Zimny et al. [22] noted that the ACL contains free nerve endings such as mechanoreceptors, Ruffini corpuscles, and Pacini corpuscles. These authors reported that the larger Ruffini corpuscles resemble Golgi corpuscles and considered them a variant of the Ruffini corpuscle. Lee et al. [13] reported that Ruffini corpuscles and Golgi corpuscles both have free nerve endings. Bali et al. [2] and Dhillon et al. [6] found Pacini corpuscles to be fusiform and that Pacini, Ruffini, and Golgi corpuscles all have free nerve endings [2, 6]. In this study, normal ACL specimens showed Pacini corpuscles and Ruffini corpuscles in the subsynovium, although the free nerve endings were extremely small (<1 μm) and non-myelinated.

Many previous studies have used the gold chloride method to visualize mechanoreceptors in ACLs. Recently, however, immunofluorescent and immunohistochemical methodologies using specific antigen-antibody reactions to detect nerve fibers have produced more reliable and relevant results [13]. In this study, we used immunohistochemical staining with monoclonal antibodies against S-100 and immunocytochemical assessment with monoclonal antibodies against Nestin to visualize mechanoreceptor-positive cells.

To verify the presence of Nestin-positive cells in the remnant tissue, the cells were treated with NGF, a critical factor for the growth, maintenance, and survival of sympathetic and sensory neurons. A previous study reported that NGF application enhanced the healing process of the ACL [14]. Here, we also observed increased proliferation of remnant ACL cells with NGF treatment, which strongly supported the identity of these cells as nervous cells. In addition, the protein and RNA expression of Nestin was observed in remnant ACL tissue, suggesting the possible existence of neuronal cells. The culture-expanded technique applied in this study was very effective at detecting cells at extremely low frequencies; using this technique, Nestin-positive cells were detected in six cases of remnant tissue.

However, four cases of remnant tissue, those from patients 2, 4, 5, and 9, did not show any Nestin-positive cells. One possible reason for this finding could be patient age, as patients 2, 4, and 5 were older than the other patients in this study. In the case of patient 9, who was relatively young but showed no Nestin-positive cells, the patient underwent a delayed operation after injury. Denti et al. [5] removed specimens from ruptured ACLs at arthroscopy but detected mechanoreceptors only in the initial few months after injury. Thus, a long delay may result in the lack of Nestin-positive cells in remnant ACL tissue. A previous study using immunohistochemistry reported that Achilles allografts do not contain newly ingrown mechanoreceptors [10]. However, in our culture-expanded ACL cells and immunocytochemical study using an anti-Nestin antibody, we demonstrated the possible existence of neuronal cells. Because Nestin is an intermediate filament protein expressed in dividing cells during the early stages of development in the central nervous system and peripheral nervous system, the existence of Nestin-positive cells in ACL remnant tissue provides strong evidence for the presence of neuronal cells.

## Conclusions

The presence of mechanoreceptors in both remnant ACL tissue and allografts was verified. Moreover, our immunocytochemical methodology proved useful. Although mechanoreceptors were detected less frequently than expected, this finding does not negate the need for remnant-preserving ACL reconstruction. Indeed, the presence of mechanoreceptors is most important, and this should not be obscured by the limitations of current detection methodologies. Together, our results support the potential regeneration of remnant mechanoreceptors, although further research is required to address the consequences of regeneration on clinical outcome.

## Abbreviations

ACL: Anterior cruciate ligament
BSA: Bovine serum albumin
DAPI: 4,6-Diamidino-2-phenylindole
ECL: Enhanced chemiluminescence
MCL: Medial collateral ligament
NGF: Nerve growth factor
PBS: Phosphate-buffered saline
ROM: Range of motion
SDS: Sodium dodecyl sulfate
